# Clinical outcomes after primary prevention defibrillator implantation are better predicted when the left ventricular ejection fraction is assessed by cardiovascular magnetic resonance

**DOI:** 10.1186/s12968-020-00640-0

**Published:** 2020-06-25

**Authors:** Laure Champ-Rigot, Pauline Gay, Frédéric Seita, Leila Benouda, Remy Morello, Arnaud Pellissier, Joachim Alexandre, Eric Saloux, Paul Milliez

**Affiliations:** 1 Service de Cardiologie, EA4650 (Signalisation, électrophysiologie et imagerie des lésions d’ischémie-reperfusion myocardique), Normandie Univ, UNICAEN, CHU de Caen Normandie, 14000 Caen, France; 2 Service de Cardiologie, Normandie Univ, UNICAEN, CHU de Caen Normandie, 14000 Caen, France; 3 Service de Radiologie, Normandie Univ, UNICAEN, CHU de Caen Normandie, 14000 Caen, France; 4 Unité de Biostatistiques et recherche clinique, Normandie Univ, UNICAEN, CHU de Caen Normandie, 14000 Caen, France; 5 Service de Pharmacologie, EA4650 (Signalisation, électrophysiologie et imagerie des lésions d’ischémie-reperfusion myocardique), Normandie Univ, UNICAEN, CHU de Caen Normandie, 14000 Caen, France

**Keywords:** Cardiovascular magnetic resonance imaging, Echocardiography, Left ventricular ejection fraction, Late gadolinium enhancement, Implantable cardioverter defibrillator, Primary prevention

## Abstract

**Background:**

The left ventricular ejection fraction (LVEF) is the key selection criterion for an implanted cardioverter defibrillator (ICD) in primary prevention of sudden cardiac death. LVEF is usually assessed by two-dimensional echocardiography, but cardiovascular magnetic resonance (CMR) imaging is increasingly used. The aim of our study was to evaluate whether LVEF assessment using CMR imaging (CMR-LVEF) or two-dimensional echocardiography (2D echo-LVEF) may predict differently the occurrence of clinical outcomes.

**Methods:**

In this retrospective study, we reviewed patients referred for primary prevention ICD implantation to Caen University Hospital from 2005 to 2014. We included 173 patients with either ischemic (*n* = 120) or dilated cardiomyopathy (*n* = 53) and who had undergone pre-ICD CMR imaging. The primary composite end point was the time to death from any cause or first appropriate device therapy.

**Results:**

The mean CMR-LVEF was significantly lower than the mean 2D echo-LVEF (24% ± 6 vs 28% ± 6, respectively; *p* < 0.001). CMR-LVEF was a better independent predictive factor for the occurrence of the primary composite endpoint with a cut-off value of 22% (Hazard Ratio [HR] = 2.22; 95% CI [1.34–3.69]; *p* = 0.002) than 2D echo-LVEF with a cut-off value of 26% (HR = 1.61; 95% CI [0.99–2.61]; *p* = 0.056). Combination of the presence of scar with CMR-LVEF< 22% improved the predictive value for the occurrence of the primary outcome (HR = 2.58; 95% CI [1.54–4.30]; *p* < 0.001). The overall survival was higher among patients with CMR-LVEF≥22% than among patients with CMR-LVEF< 22% (*p* = 0.026), whereas 2D echo-LVEF was not associated with death.

**Conclusions:**

CMR-LVEF is better associated with clinical outcomes than 2D echo-LVEF in primary prevention using an ICD. Scar identification further improved the outcome prediction. The combination of CMR imaging and echocardiography should be encouraged in addition to other risk markers to better select patients.

## Background

Randomized controlled trials have proven the benefit of implanted cardioverter defibrillator (ICD) therapy in patients with altered left ventricular ejection fraction (LVEF) in both secondary and primary prevention of sudden cardiac death [1–7]. The only reliable criterion to select candidates for primary prevention ICD is the severity of LVEF impairment. Two-dimensional echocardiography (2D echo) is widely used despite several limitations, particularly high intra and inter observer variability. Moreover, Simpson’s biplane method is based on geometric assumptions inconsistent with wall deformations occurring in dilated failing ventricles; however, cardiovascular magnetic resonance (CMR) imaging is more accurate [8] and is considered the non-invasive gold standard for LVEF assessment [9]. Nevertheless, current guidelines recommend ICD implantation for patients with LVEF < 35% regardless of the imaging method [10, 11]. Late gadolinium enhancement (LGE) CMR imaging also allows scar identification known as the malignant arrhythmia substrate [12].

The aim of our study was to evaluate whether LVEF assessment with either CMR imaging (CMR-LVEF) or echocardiography (2D echo-LVEF) may predict differently the occurrence of clinical outcomes among patients referred for primary prevention ICD implantation.

## Methods

We retrospectively identified patients referred to Caen University Hospital for primary prevention ICD implantation from January 2005 to December 2014. We included patients who had undergone both 2D echo and CMR imaging less than 3 months prior to ICD implantation. All the patients met the implantation criteria according to the guidelines currently used during the inclusion period [13, 14]: patients with ischemic cardiomyopathy (ICM) or nonischemic cardiomyopathy (NICM) in New York Heart Association (NYHA) class II or III and LVEF ≤35%; before the updated guidelines in 2008, patients with ICM and LVEF < 30% regardless of NYHA class. They were all stable and under optimal medical therapy. The exclusion criteria were as follows: ICD implantation for another structural heart disease (e.g., hypertrophic cardiomyopathy, arrhythmogenic cardiomyopathy, infiltrative cardiomyopathy or inherited primary arrhythmia syndromes); extended delay (> 90 days) between 2D echo and CMR; a significant clinical event (i.e., hospitalization, atrial fibrillation, and cardiac rehabilitation) between each LVEF assessment or before implantation; poor 2D echo imaging quality with nonreliable LVEF assessment. In the case of LVEF mismatch, defined as LVEF ≤35% with one imaging modality but > 35% with the other, ICD implantation was decided after team consensus. Demographic data and clinical features from the patients enrolled were anonymously collected from medical records. The MAGGIC (Meta-analysis global group in chronic heart failure) risk score was calculated in all patients [15].

### Echocardiography

Echocardiograms were acquired by two experienced sonographers using either SONOS 5500® (Philips Healthcare, Amsterdam, Netherlands) or IE33® (Philips Healthcare) with a 3.5-MHz probe. Three cardiac cycles were recorded in sinus rhythm and ten in the case of atrial fibrillation. In the case of poor image quality, ultrasound contrast agent (Sonovue®; Bracco International BV, Milan, Italy) was injected. The data were reviewed and analyzed offline using commercially available software (Xcelera®; Philips Healthcare) by two investigators blinded to any clinical or imaging data. 2D echo-LVEF was computed from the left ventricular (LV) end-diastolic volume and end-systolic volume using the biplane Simpson’s method on two- and four-chamber apical views. The endocardial border was manually delineated according to the American Society of Echocardiographic recommendations [16].

### CMR data acquisition

CMR imaging was performed with a 1.5 T CMR unit (Signa Excite HDxt General Electric Healthcare, Waukesha, Wisconsin, USA). with a 5-element phased-array body coil. Electrocardiographically gated localizing sequences were used to identify the long axis of the heart to allow imaging of the LV in the anatomically correct short-axis plane. The acquisition protocol included several sequences that covered the LV from the base to the apex. Cine images were acquired for 10- to 15-s breath-holds with a temporal resolution of 30 frames/cardiac cycle. The balanced steady-state free precession (bSSFP) cine sequence scanning parameters were: 7 mm thickness, 1 mm gap, TR = 3.9 ms, TE = 1.7 ms, flip angle 60°, matrix 256 × 256, FOV 40 cm. LGE bidimensional sequences were acquired 10–15 min after bolus injection of gadoterate meglumine (Dotarem™ 0.15 mmol/kg, Guerbet, Aulnay-sous-Bois, France) with the appropriate parameters: 8 mm thickness, 1 mm gap, TR = 6.7 ms, TE = 3.1 ms, flip angle 20°, matrix 256 × 192, FOV 40 cm. An inversion time scout sequence was used to select an inversion time between 200 and 300 ms for optimal nulling of normal myocardium. In the case of atrial fibrillation, a mean representative cycle was selected, or, if needed, a real time imaging sequence (MR Echo®, General Electric Healthcare) was used in short axis (8 mm thickness, 1 mm gap, TR = 3.5 ms, TE = 1.5 ms, flip angle 20°, matrix 128 × 72, FOV 36 cm).

### CMR images post-processing and data analysis

CMR analyses were performed by experienced radiologist and cardiologist blinded to all clinical and imaging data using a cardiac workstation with ReportCard 2.0 software (General Electric Healthcare). Short-axis cine images were used to determine the LV end-diastolic volume, end-systolic volume, and LVEF. The epicardial and endocardial contours of the LV myocardium were then defined manually by including the papillary muscles in the cavity by cine images [17]. Scar tissue was defined as an area of tissue with an increased signal intensity visually detected, in ≥2 perpendicular views using LGE images, with a binary approach (scar tissue vs. normal myocardium).

### ICD implantation

All the patients underwent ICD implantation according to the standard surgical technique. Patients who met the required criteria (NYHA class ≥2 associated with left-bundle branch block QRS morphology and QRS duration> 120–130 ms or non-left bundle branch block QRS morphology and QRS duration > 150 ms) were implanted with a cardiac resynchronization therapy defibrillator. All manufacturers were represented in our population: Saint Jude Medical (Saint Paul, Minnesota, USA), Medtronic (Minneapolis, Minnesota, USA), Biotronik (Berlin, Germany), Boston Scientific (Malborough, Massachusetts, USA), and Sorin Group (Clamart, France). ICD devices were programmed using two or three zones: an optional monitoring zone without therapy, a ventricular tachycardia zone (> 170/min) with anti-tachycardia pacing followed by shocks and a ventricular fibrillation (> 214/min) zone with shocks.

### Clinical follow up and end points

Patient follow up was conducted according to our standard of care with clinical examination and device interrogation. It was performed before hospital discharge, at 1 month and every 6 months. The patients were given a remote monitoring system, if available, and were followed as other patients every 6 months. Appropriate device therapy (ADT) was defined as an episode of anti-tachycardia pacing and/or internal shocks delivered to terminate ventricular arrhythmia as previously defined. All ADTs were confirmed by an electrophysiologist who analyzed device reports and electrograms. Inappropriate therapies were defined as anti-tachycardia pacing and/or internal shocks delivered in the case of supraventricular tachycardia, T-wave detection, noise (i.e., electromyogram) or lead failure. The primary end point was defined as the occurrence of the first composite event including death or ADT. Secondary end points were all-cause mortality and the occurrence of the first ADT.

### Statistical analysis

Continuous variables were expressed as the means ± standard deviation (SD) if normally distributed and as medians and interquartile range if not normally distributed. Categorical variables were expressed as numbers and percentages. Continuous data were compared using Student’s *t-*test or the Mann-Whitney test as appropriate. Comparisons between categorical or dichotomous data were performed using chi-square test or Fisher’s exact test depending on validity criteria. Reproducibility between the imaging techniques was performed using Bland and Altman analysis. Survival time until the first ADT or death was defined as the number of days between implantation to the first event. If a patient did not experience any end point during the follow-up period, the outcome was considered censored. Univariate and multivariate Cox regression analyses were used to examine the association between the baseline characteristics and occurrence of the first ADT or all-cause mortality. Each variable with a *p* value < 0.15 in univariate analysis was introduced in multivariate analysis. The assessment of 2D echo-LVEF and CMR-LVEF threshold values to predict the occurrence of ADT or mortality was realized using the receiver operating characteristic (ROC) curve method. Survival analysis and comparisons between subgroups defined using LVEF thresholds were evaluated using Kaplan-Meier estimates and the log-rank test. All statistical analyses were performed using IBM SPSS Statistics, version 20.0 (released 2011; Statistical Package for the Social Sciences, International Business Machines, Inc., Armonk, New York, USA). A *p* value < 0.05 was considered statistically significant.

## Results

**Fig. 1:**
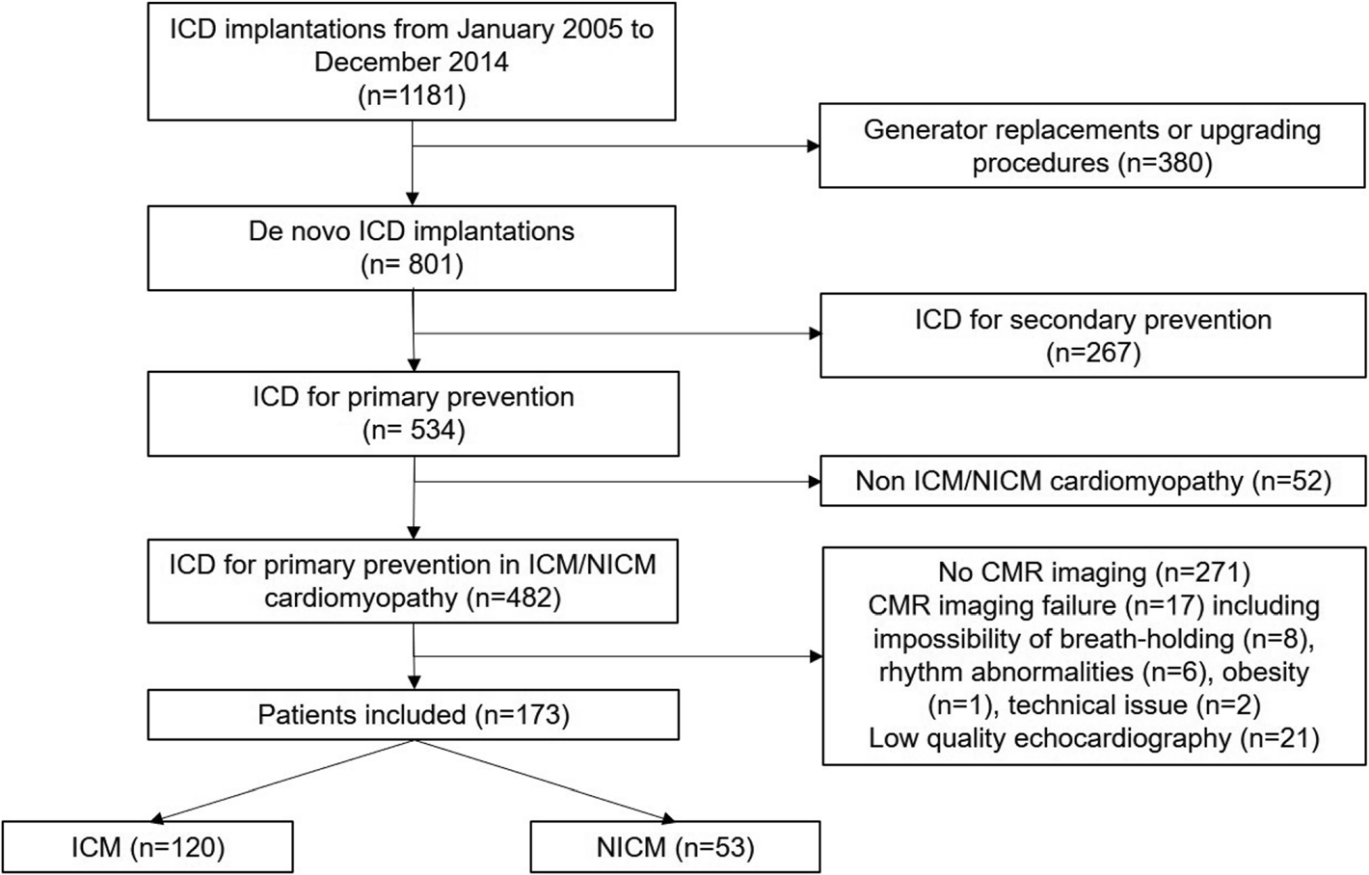
Study flow chart. CMR: cardiovascular magnetic resonance; ICD: implanted cardioverter defibrillator; ICM: ischemic cardiomyopathy; NICM: nonischemic cardiomyopathy

During the study period, 1181 patients were successfully implanted with an ICD at Caen University Hospital, including 534 with ICM or NICM referred for primary prevention of sudden cardiac death. One hundred seventy-three of them underwent both preimplantation CMR imaging and 2D echo; therefore, they were included in our study (Fig. 1).

The mean age was 59 ± 12 years; the patients were mostly men (86%), with ICM in 69%. Most of them had mild dyspnea and were in NYHA II class. Medical therapy was optimized with 94% of patients receiving a beta-blocker, 94% receiving a renin-angiotensin system blocker and 61% receiving a mineralocorticoid receptor antagonist. The mean QRS duration was 124 ± 34 milliseconds, and 32% of the patients received a resynchronization device. The baseline characteristics are all summarized in Table 1. During the mean follow up of 50 ± 30 months, death occurred in 46 patients (26.6%). We observed ADT in 30 patients (17.3%) and seven inappropriate therapies (4%).

**Table 1:** Characteristics of the patients at baseline and according to the primary end point occurrence

	*N* = 173	Death or ADT		p	p for survival
		Yes (*n* = 66)	No (*n* = 107)		
Follow-up, months (mean ± SD)	50,3 ± 30,3	52,5 ± 28,7	46,7 ± 32,7	0,223	
Age, years (mean ± SD)	59 ± 12	60 ± 13	58 ± 11	0,554	(≥ vs < 58) 0,979
Male, n (%)	149 (86)	62 (94)	87 (81)	0,023	0,206
BMI, kg/m^2^ (mean ± SD)	27.2 ± 5	27.9 ± 4	26.9 ± 5	0.476	(≥ vs < 27) 0.730
ICM, n (%)	120 (69)	54 (82)	66 (62)	0,006	0,046
Revascularization, n(%)					
PCI	74 (62)	33 (59)	41 (64)	0.132	0.026
CABG	14 (12)	10 (18)	4 (6)		
None	32 (27)	13 (23)	19 (30)		
Diabete mellitus, n (%)	40 (23)	14 (21)	26 (24)	0,712	0,819
Hypertension, n (%)	49 (28)	24 (36)	25 (23)	0,082	0,046
Smokers, n (%)	64 (37)	28 (42)	36 (34)	0,260	0,918
COPD (Gold ≥2), n (%)	18 (10)	10 (14)	8 (8)	0.203	0.316
Cancer, n (%)	4 (2.3)	2 (3)	2 (2)	1	0.299
AF, n (%)	35 (20)	20 (30)	15 (14)	0,012	0,196
NYHA class, n (%)					
I	28 (16)	9 (14)	19 (18)	0,270	II vs I: 0,508
II	99 (57)	35 (53)	64 (60)		III vs I: 0,079
III	46 (27)	22 (33)	24 (22)		III vs I + II: 0,054
Heart rate, ppm (mean ± SD)	69 ± 16	66 ± 13	71 ± 18	0,108	0,354
QRS duration, ms (mean ± SD)	124 ± 33	121 ± 32	125 ± 34	0,413	0,384
eGFR, ml/min (mean ± SD)	67.4 ± 20.3	59.5 ± 17.7	67.4 ± 19.9	0.008	(≥ vs < 60) 0.932
CKD 3b, n (%)	25 (15)	15 (22)	10 (10)	0.03	0.316
Betablockers, n (%)	163 (94)	64 (97)	99 (93)	0,321	0,270
ARB or ACEI, n (%)	163 (94)	62 (94)	101 (94)	1000	0,661
Diuretics, n (%)	124 (72)	47 (71)	77 (72)	1000	0,939
MRA, n (%)	105 (61)	41 (62)	64 (60)	0,873	0,556
Resynchronization therapy, n (%)	56 (32)	21 (32)	35 (33)	1000	0,978
MAGGIC Score (mean ± SD)	20 ± 6	22 ± 7	19 ± 5	0.040	(≥ vs < 19) 0.105
Sodium, mmol/l (mean ± SD)	138 ± 3	137 ± 3	138 ± 3	0.071	(≥ vs < 138) 0.920
BNP, pg/ml (mean ± SD)	625 ± 622	674 ± 511	592 ± 689	0.449	(≥ vs < 400) 0.462
Hematocrit, % (mean ± SD)	40 ± 19	39 ± 4	41 ± 4	0.042	(≥ vs < 41) 0.035
2D echo-LVEF, % (mean ± SD)	27,5 ± 6,3	27 ± 6	28 ± 7	0,500	0,256
2DEcho-LVEF< 26, n (%)	63 (36)	30 (46)	33 (33)	0,141	0,052
CMR-LVEF, % (mean ± SD)	23,4 ± 6,7	22 ± 7	24 ± 7	0,087	0,010
CMR-LVEF< 22, n (%)	60 (35)	28 (42)	32 (30)	0,102	0,005
LGE, n(%)	133 (77)	58 (87)	75 (71)	0,017	0,058
LGE and CMR-LVEF< 22, n(%)	44 (25)	26 (39)	18 (18)	0,002	< 0,001
LGE and 2D echo-LVEF< 26, n(%)	50 (29)	28 (42)	22 (22)	0,010	0,010
Complications, n (%)	23 (13)	9 (14)	14 (13)	1000	0,695
Infections, n (%)	7 (4)	1 (1)	6 (6)	0.245	0.702
Other complications	16 (9)	8 (1)	8 (7)		
Inappropriate device therapies, n(%)	7 (4)	7 (10)	0 (0)	0.001	0.730

Continuous variables are normally distributed, expressed as mean and standard deviation. AF: atrial fibrillation; *ACEI* angiotensine converting enzyme inhibitor; *ARB* angiotensine receptor blockers; *BMI* body mass index; *BNP* brain natriuretic peptide; *CABG* coronary artery bypass graft; *CKD 3b* chronic kidney disease stage 3b defined by eGFR< 45 ml/min; *CMR-LVEF* cardiovascular magnetic resonance left ventricular ejection fraction; *COPD* chronic obstructive pulmonary disease; *eGFR* estimated glomerular filtration rate; *ICM* ischemic cardiomyopathy; *LGE* Late gadolinium enhancement; *MAGGIC* meta-analysis global group in chronic heart failure; *MRA* Mineralocorticoid receptor antagonist; *NYHA* New-York Heart Association; *ppm* pulse per minute; *PCI* percutaneous coronary intervention; *SD* standard deviation; *2DEcho-LVEF* two dimensional echocardiography left ventricular ejection fraction

### LVEF measurement with echocardiography and CMR imaging

**Fig. 2:**
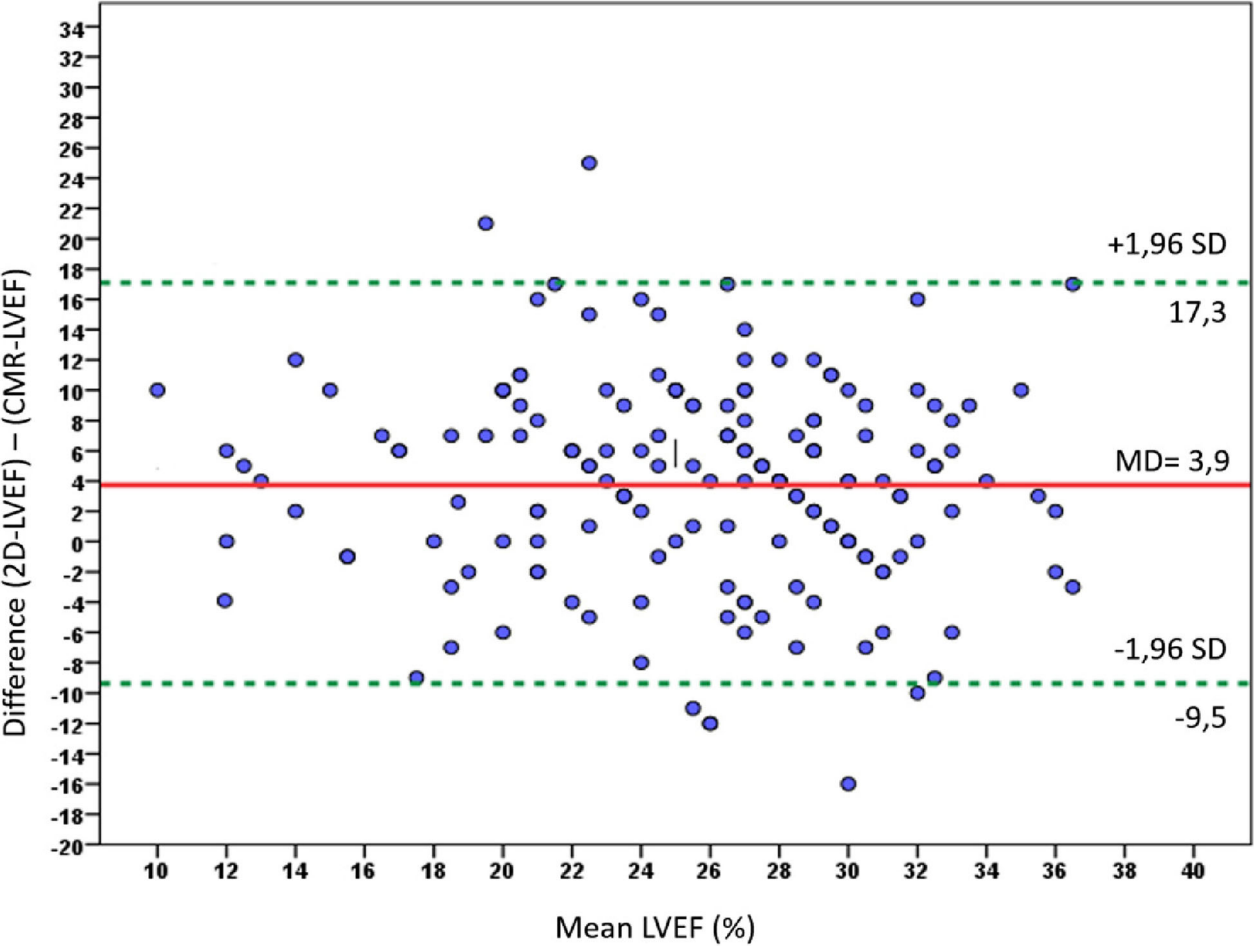
Bland and Altman plots of differences in left ventricular ejection fraction (LVEF) between two dimensional (2D) echocardiography (2DEcho-LVEF) and cardiovascular magnetic resonance (CMR). The red line represents the mean paired difference, and the green dotted lines represent the 95% limits of agreement. MD: mean difference; SD: standard deviation

At the time of implantation, the mean CMR-LVEF (24 ± 6%) was significantly lower than the mean 2D echo-LVEF (28 ± 6%) (*p* < 0.001). The intraclass correlation coefficient between 2D echo-LVEF and CMR-LVEF was 0.466 (p < 0.001). Figure 2 represents the mean difference between 2D echo-LVEF and CMR-LVEF for each patient according to the Bland and Altman method. The overall mean difference between 2D echo-LVEF and CMR-LVEF was 4 ± 7%. The limits of agreement were wide between the two methods (− 9.5 to 17.3).

### Occurrence of death or appropriate device therapy

The primary end point, death from any cause or the first ADT, occurred in 66 patients (38%) during a mean follow-up of 50.3 ± 30.3 months. Ten patients received ADT before death and were censored for primary end point analysis. There was no difference between patients who met the primary end point regarding age, sex ratio or treatment. There was a trend toward a higher risk of meeting the primary end point for patients in NYHA class III than for patients in NYHA class I or II (*p* = 0.054). ICM was significantly associated with death or ADT (81.8% vs 61.7%, *p* = 0.006) and correlated with survival (*p* = 0.046). Among these latter, those who had no revascularization had the worse survival free from the primary endpoint (log-rank *p* = 0.026). A higher creatinine level (*p* = 0.005) and a lower hematocrit (*p* = 0.042) were also found to be associated with the occurrence of death or ADT. The MAGGIC score was higher in patients who experienced the primary outcome (p = 0.04). The characteristics of patients according to the occurrence of the primary end point are summarized in Table 1.

#### Primary endpoint prediction using 2D echo LVEF and CMR LVEF

**Fig. 3:**
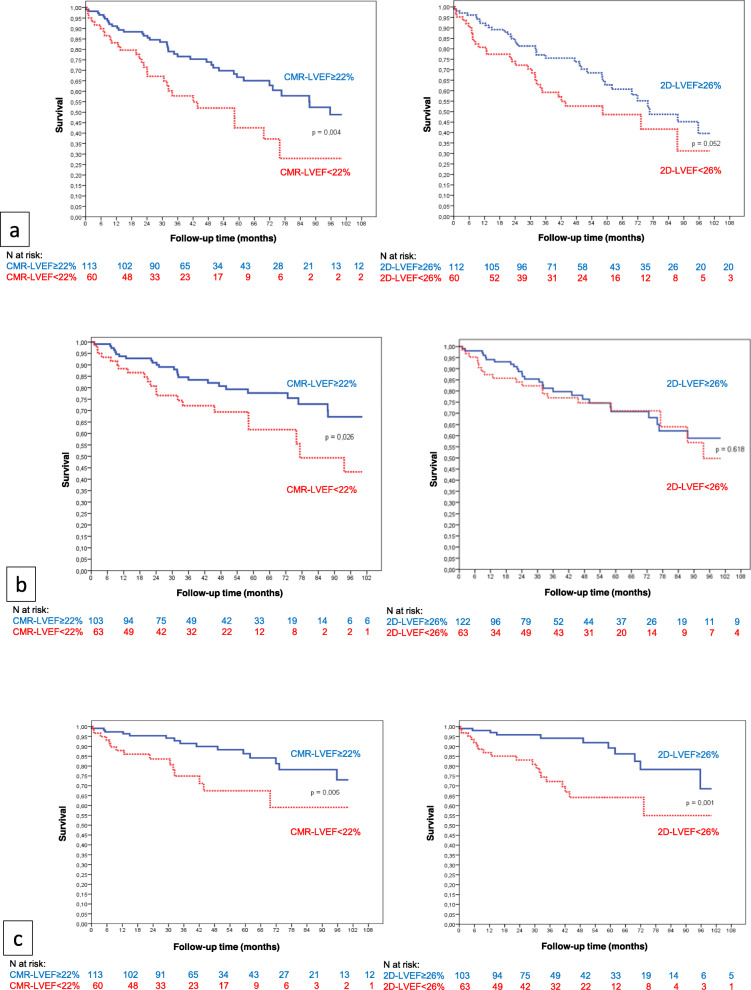
Survival curves based on LVEF assessed by CMR-LVEF and 2DEcho-LVEF). **a** Primary composite end point of death or appropriate device therapy; **b** Death; **c** Appropriate device therapy

Patients who died or received ADT had lower CMR-LVEF (22 ± 7%) than those who did not (24 ± 7%) (*p* = 0.01), whereas the mean 2D echo-LVEF was not different between those patients. We identified LVEF threshold values to stratify patients with a higher risk of death or ADT by ROC curve analysis. Event-free survival was greater among the patients with CMR-LVEF≥22% (*p* = 0.04). After Cox regression, we found that CMR-LVEF< 22% was associated with a 2.22 greater risk of death or ADT during follow-up (95% CI [1.34–3.69]; *p* = 0.002). There was a trend toward better survival among patients with 2D echo-LVEF≥26% (*p* = 0.052), and 2D echo-LVEF< 26% was associated with a 1.61 higher risk of meeting the primary end point (95% CI [0.99–2.61]; *p* = 0.056) (Fig. 3, panel a). Among the 120 patients with ICM, the mean CMR-LVEF was significantly lower in the case of death or ADT (22% versus 25%, *p* = 0.027). There was also a trend toward a lower 2D echo-LVEF when the primary end point occurred (27% versus 29%, *p* = 0.088). Conversely, neither CMR nor 2D echo-LVEF were associated with the primary end point occurrence in patients with NICM (*p* = 0.836 and 0.858 respectively).

#### Scar identification combined with LVEF

**Fig. 4:**
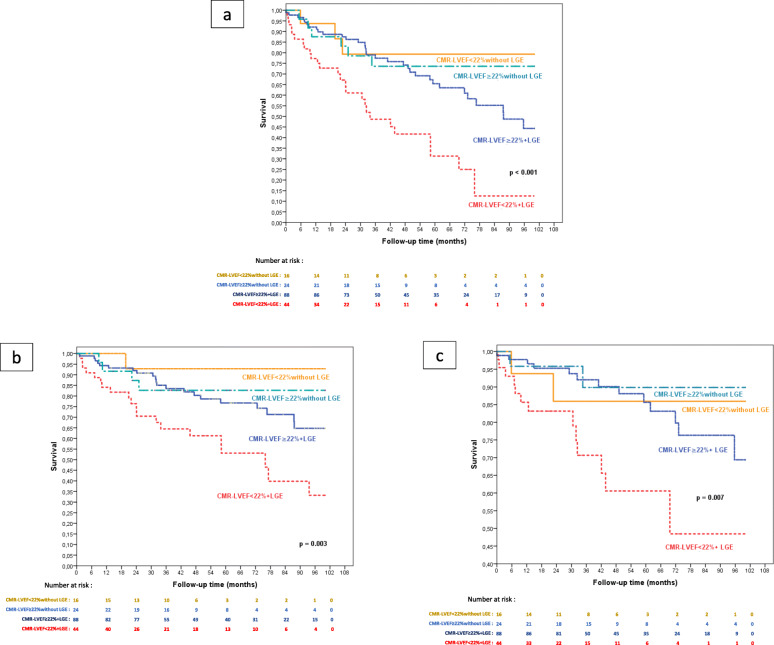
Survival curves based on the combination of CMR-LVEF and presence of late gadolinium enhancement (LGE). **a** Primary composite end point of death or appropriate device therapy; **b** Death; **c** Appropriate device therapy

In our study population, we found LGE in 133 patients out of 173 (76.9%). A large number of patients with ICM had LGE (116/120), whereas myocardial scar was found in fewer patients presenting with NICM (17/53). LGE presence was significantly associated with the primary outcome (*p* = 0.017). Survival free from the primary outcome was significantly lower in patients with both CMR-LVEF< 22% and LGE compared to those with CMR-LVEF≥22% and LGE, and those who had no LGE regardless of CMR-LVEF (log-rank *p* < 0.001). Survival curves for the occurrence of death and ADT also showed a significant impact of the combination of CMR-LVEF< 22% and scar presence. Survival curves based on CMR-LVEF and LGE presence are represented in Fig. 4. We combined LGE and LVEF thresholds as previously described and found improvement in the outcome prediction. The hazard ratio (HR) associated with CMR-LVEF< 22% and LGE presence was 2.58 (95% CI [1.54–4.30]; *p* < 0.001) for the primary combined outcome.

### Mortality

At the end of the follow up, 46 patients died (26.6%). NYHA class III (*p* = 0.002), atrial fibrillation (*p* = 0.02) and a high level of plasmatic creatinine (*p* = 0.001) were associated with the risk of death in our study. The overall survival was higher among patients with CMR-LVEF≥22% than among patients with CMR-LVEF< 22% (*p* = 0.026). CMR-LVEF< 22% was associated with the risk of death in both univariate and multivariate analyses with a 2.02 HR (95% CI [1.1–3.69]; p = 0.02). The HR associated with CMR-LVEF< 22% and LGE presence was 3.26 (95% CI [1.56–6.86]; *p* = 0.002) for mortality. By contrast, the mean 2D echo-LVEF was not different in patients who died compared with the others and the previous 2D echo-LVEF threshold was not associated with the overall survival (Fig. 3, panel b).

### Appropriate device therapy

During the follow up, 30 patients (17%) experienced ADT. We found no clinical characteristic predictive of ADT occurrence Conversely, survival free from ADT was significantly higher for patients with CMR-LVEF≥22% (*p* = 0.005) or 2D echo-LVEF≥26% (*p* = 0.001) (Fig. 3, panel c). The HR associated with CMR-LVEF< 22% and LGE presence was 2.70 (95% CI [1.49–4.87], p = 0.001) for ADT occurrence.

### CMR and 2D Echo mismatch

Among the patients, 17 had discordant LVEF measurements between CMR imaging and 2D echo. Eleven had CMR-LVEF≤35% but 2D echo-LVEF> 35% and would have not received an ICD in the case of echocardiography-based screening. Two of them died, and three experienced ADT. Conversely, six patients had 2D echo-LVEF≤35% but CMR-LVEF> 35%; two of these died, and none had ADT. Despite a too small sample size to conclude, patients with LVEF mismatch did not seem to have worse outcomes compared to the rest of the cohort.

## Discussion

Our report, studying patients implanted with ICD for primary prevention according to the current guidelines, showed the following: CMR imaging and 2D echo exhibited only moderate agreement to assess LVEF and 2D echo-LVEF was found to be higher than CMR-LVEF; CMR-LVEF was better associated with death or ADT than 2D echo-LVEF despite close threshold values; LGE presence improved the predictive value of CMR-LVEF.

### Discrepancies between 2D echo-LVEF and CMR-LVEF

Several reports have shown that CMR imaging was superior to 2D echo and more reproducible for LVEF assessment [18]. Bellenger et al. [8] suggested in a direct comparison between these two modalities and radionuclide ventriculography that CMR imaging should be preferred regarding its volumetric approach and high image quality. Particularly, at lower LVEF, 2D echo generally overestimates LVEF compared with CMR imaging [8, 19, 20]. We found a systematic bias of 4 ± 7% between 2D echo-LVEF and CMR-LVEF. Higher LVEF measurements by 2D echo compared with CMR was already pointed out by Rijnierse et al. [21] who found a mean difference of 6 ± 7%. In daily practice, a five-percent difference in LVEF could be considered non relevant. However, because the guidelines are based on the 35% cut-off value, the choice of either 2D echo-LVEF or CMR-LVEF could result in the reclassification of patients for ICD eligibility. This issue affects patients with borderline LVEF. Indeed, having a 2D echo-LVEF within 5 % of commonly used thresholds was found to be predictive of reclassification by CMR for ICD implantation [19].

### CMR-LVEF and cardiac events

Some reports have already shown that using the same threshold value with CMR imaging and 2D echo would result in a greater number of patients to implant with CMR imaging [19–22]. In a retrospective study including patients selected for ICD according to CMR-LVEF≤35%, Rijnierse et al. showed that 19% of patients would not have been implanted according to 2D echo-LVEF. These patients had a significantly lower rate of ADT than those with 2D echo-LVEF< 35% [21]. Conversely, Rayatzadeh et al. found in their primary prevention population selected according to 2D echo-LVEF that 25% of the patients did not meet ICD eligibility based on CMR imaging. Those patients experienced no appropriate therapy. Despite their patients were a little older than ours, they were quite comparable regarding baseline clinical characteristics. They also reported that survival free from ADT was stratified by an empirical 30% CMR-LVEF cut-off value, whereas the same value of 2D echo-LVEF did not predict ADT among patients [22]. Based on different data, they both suggested to use a different and lower value for CMR-LVEF to better assess ICD eligibility. To further explore this hypothesis, we did not compare the eligibility according to the imaging method but sought to determine both 2D echo and CMR-LVEF thresholds associated with cardiac outcomes among patients with primary prevention ICD therapy, that constitutes the novelty of our work compared to previous reports. Finally, we found that the LVEF cut-off values to predict death or ADT were very low, either with CMR imaging or 2D echo and much lower than the recommended 35% cut-off. It is worth noting that the same comments were addressed to the large randomized trials that included patients with lower LVEF than the inclusion criteria requirements. The mean LVEF in those studies was 20 to 25% [1, 4, 5, 23], and the European guidelines committee already warned about inconsistencies in recommendations based on these results [13]. The average LVEF differences between patients with events (death or ADT) and others were low in our study (< 5%). Nevertheless, one of the major findings of our work is that the CMR-LVEF threshold seemed better correlated with the clinical outcomes than 2D echo-LVEF in a real-life population of primary prevention patients. The overall survival was stratified by the CMR-LVEF threshold, whereas the 2D echo-LVEF cut-off value was only associated with the occurrence of ADT. Recently, the DANISH trial showed that prophylactic ICD implantation was not associated with better survival in nonischemic patients [24]. Our study population, especially the NICM group, was too small to draw any conclusion. However, Gao et al. have already shown that patients with ICM who experienced ADT or survived cardiac arrest or sudden cardiac death had significantly lower CMR-LVEF than those without events, whereas LVEF was not different in cases of NICM [25]. Although underpowered, our results are consistent with previous reports suggesting that LVEF alone might not be sufficient to select ICD candidates with NICM.

### Additional predictive value of LGE combined with CMR-LVEF

Several studies have reported that LGE presence and extent were associated with ventricular arrhythmias in both ICM [26] and NICM [27]. A meta-analysis of 19 studies reported a pooled odds ratio of 5.62 for ventricular arrhythmias in patients with LGE above study-defined thresholds with no difference between ICM and NICM subgroups [28]. Recently, a prospective study conducted in NICM patients reported a reduction in mortality with primary prevention ICD implantation only in patients presenting a LV scar [29]. We showed that the combination of CMR-LVEF< 22% and LGE presence improved the prediction of death or ADT in primary prevention patients with a 2.58 HR (*p* < 0.001) over CMR-LVEF alone (HR = 2.22; *p* = 0.002). Pontone et al. also compared the prognostic benefit of CMR-LVEF associated with the presence of LGE over 2D echo-LVEF for the evaluation of 409 potential candidates to primary prevention ICD therapy. Their patients had higher LVEF and were older than ours, only 34% of them were finally implanted with an ICD and they were compared with the acknowledged 35% LVEF threshold. Nevertheless, the authors also highlighted that CMR-LVEF provided additional discriminatory value to the assessment of LVEF by 2D echo alone, with a further improvement when combining CMR-LVEF depression and the presence of LGE to predict cardiac rhythmic events [30].

### Possible clinical implications

Currently, there is no other validated criterion to select patients for ICD-based primary prevention than LVEF. To better identify patients who will derive a benefit from ICD, there is growing evidence supporting CMR imaging use. Our study highlighted the CMR ability to identify patients with a higher risk of death or ventricular arrhythmias. CMR-LVEF alone was better associated with cardiac outcomes than 2D echo-LVEF, and the combination with myocardial scar presence further improved its predictive value. When available, CMR imaging can be useful in addition to 2D echo, especially for challenging situations—i.e., patients with borderline LVEF or NICM. Future studies will be needed to find a dedicated CMR-LVEF threshold, probably lower than 35%, to select patients for primary prevention ICD. Several features (LVEF, right ventricular function, scar presence, NYHA class etc.) are associated with the risk of death and arrhythmias, but except the alteration of LVEF there is no other validated criteria to select patients. It could be at particular interest to develop and validate a risk model that should integrate CMR data (LVEF, LGE presence, location, extent) and clinical features like NYHA class or T-waves alternance. Further studies should determine the weight of each item and a score associated with the benefit of ICD implantation.

### Limitations

This study was single-center and retrospective with a small sample size, especially the NICM subgroup, limiting definitive conclusions. CMR imaging prior to implantation occurred at the physician’s discretion. At our center, as in other tertiary centers, access to this imaging modality is not easy to organize, especially for patients referred from other centers in our area. Numerous patients referred for primary prevention at our center were not evaluated with CMR imaging and some others had CMR because of low-quality 2D echo or had uninterpretable CMR study (see flow chart). We examined the excluded patients regarding baseline characteristics age, gender, risk factors, type of cardiomyopathy, NYHA status, medications and LVEF prior to implantation (see supplemental table). There was no difference in LVEF, QRS duration, the sinus rhythm rate, and the creatinine level. Nevertheless, excluded patients were older, more symptomatic, had more hypertension, NICM, and received more CRT device. This last difference is likely to explain previous ones. They also had a higher MAGGIC score than the included patients. This selection bias may limit the external validity of our results because the excluded patients were in more severe condition with a higher risk of cardiac events. Nevertheless, in our study population, who was at lower risk of events, we were able to show the interest of the CMR assessment of LVEF combined with LGE in predicting death and/or ADT. We could assume that the interest of CMR to predict events should be maintained in a higher risk population. Even though this issue of external validity should be addressed by prospective studies/registries. We also did not retrieve sufficient three-dimensional echocardiography data to compare with 2D echo and CMR imaging. 2D echo and CMR imaging were not realized at the same time, and we could assume that LVEF modification might occur. However, there was fewer than 3 months between them and patients experiencing a cardiac event during this period were excluded. Despite a possible important clinical implication, the “mismatch group” was far too small to support any conclusion about the outcomes of patients who have discordant LVEF measurements.

## Conclusions

In our population of patients selected for primary prevention ICD implantation, CMR-LVEF and 2D echo-LVEF showed poor agreement and CMR-LVEF was significantly lower. We found that CMR-LVEF was better associated with the risk of death and ADT than 2D echo-LVEF. Furthermore, LGE detection associated with a low CMR-LVEF improved cardiac outcome prediction. Routine use of CMR imaging in addition to 2D echo should be supported in patients’ evaluation for ICD in primary prevention.

## Supplementary information

**Additional file 1.** Table 1: Characteristics of patients with CMR study compared with the patients excluded (no CMR, CMR failure, low quality echocardiography).

## Data Availability

The datasets used and/or analyzed during the current study are available from the corresponding author on reasonable request.

## Abbreviations

2D: Two dimensional
ADT: Appropriate device therapy
bSSFP: Balanced steady state free precession
CI: Confidence interval
CMR: Cardiovascular magnetic resonance
Echo: Echocardiography
HR: Hazard ratio
ICD: Implanted cardioverter defibrillator
ICM: Ischemic cardiomyopathy
LGE: Late gadolinium enhancement
LV: Left ventricle/left ventricular
LVEF: Left ventricular ejection fraction
MAGGIC: Meta-analysis global group in chronic heart failure
NICM: Nonischemic cardiomyopathy
NYHA: New York Heart Association
ROC: Receiver operating characteristic
