# Supplementary material for: Clinical outcomes after primary prevention defibrillator implantation are better predicted when the left ventricular ejection fraction is assessed by cardiovascular magnetic resonance
Source: J Cardiovasc Magn Reson. 2020 Jun 25;22:48. doi: 10.1186/s12968-020-00640-0 (PMC7315498; doi:10.1186/s12968-020-00640-0)
Supplement: Supplementary file 1 — Additional file 1. Table 1: Characteristics of patients with CMR study compared with the patients excluded (no CMR, CMR failure, low quality echocardiography). [file 12968_2020_640_MOESM1_ESM.docx]

**Supplemental table 1**: Characteristics of patients with CMR study compared with the patients excluded (no CMR, CMR failure, low quality echocardiography).

|  | total | CMR | Excluded | p |
| --- | --- | --- | --- | --- |
|  | n=482 | n=173 | n=309 |  |
| Age, years (mean±SD) |  | 59 (±12) | 65 (±10) | <0,001 |
| Male gender, n(%) | 407 (84,4) | 149 (86,1) | 258 (83,5) | 0,513 |
| Hypertension, n(%) | 186 (39,9) | 49 (28,3) | 137 (46,8) | <0,001 |
| Diabetes mellitus, n (%) | 119 (25,5) | 40 (23,1) | 79 (26,9) | 0,382 |
| Creatinine level, mmol/l (mean±SD) |  | 108 (±36,0) | 113,7 (±85,3) | 0,419 |
| LVEF, % (mean±SD) |  | 27,5 (±6,3) | 27,2 (±4,8) | 0,655 |
| Sinus rhythm, n(%) | 399 (83) | 138 (79,8) | 261 (84,7) | 0,104 |
| QRS duration, ms (mean±SD) |  | 123 (±33) | 127 (±34) | 0,289 |
| NYHA, n (%) |  |  |  | 0,006 |
| 1 | 57 (11,8) | 28 (16,2) | 29 (9,4) |  |
| 2 | 258 (53,5) | 99 (57,2) | 159 (51,5) |  |
| 3 | 167 (34,6) | 46 (26,6) | 121 (39,2) |  |
| Cardiomyopathy, n(%) |  |  |  | 0,004 |
| ICM | 292 (60,6) | 120 (69,4) | 172 (55,7) |  |
| NICM | 190 (39,4) | 53 (30,6) | 137 (44,3) |  |
| Medications, n (%) |  |  |  |  |
| Beta-blockers | 457 (95) | 163 (94,2) | 294 (95,5) | 0,663 |
| ACEI/ARB | 455 (94,6) | 163 (94,2) | 292 (94,8) | 0,835 |
| MRA | 238 (49,7) | 105 (60,5) | 133 (43,5) | <0,001 |
| Loop diuretics | 367 (76,5) | 124 (71,7) | 243 (79,2) | 0,073 |
| CRT | 208 (43,2) | 56 (32,4) | 152 (49,2) | <0,001 |
| MAGGIC score, (mean±SD) | 22 ± 6 | 20 ± 6 | 23 ± 7 | 0.011 |

ACEI: angiotensin converting enzyme inhibitor; ARB: angiotensin receptor blockers; CMR: cardiovascular magnetic resonance imaging; CRT: cardiac resynchronization therapy; ICM: ischemic cardiomyopathy; LVEF: left ventricular ejection fraction; MAGGIC: meta-analysis global group in chronic heart failure; MRA: mineralocorticoid receptor antagonist; NICM: nonischemic cardiomyopathy; NYHA: New York Heart Association
